# Coronapandemie: Auswirkungen auf den Einzelhandel in den Innenstädten

**DOI:** 10.1007/s10273-022-3153-0

**Published:** 2022-04-19

**Authors:** Henry Goecke, Christian Rusche

**Affiliations:** 1 Forschungsgruppe Big Data Analytics, Institut der deutschen Wirtschaft Köln e.V., Konrad-Adenauer-Ufer 21, 50668 Köln, Deutschland; 2 Institut der deutschen Wirtschaft Köln e.V., Konrad-Adenauer-Ufer 21, 50668 Köln, Deutschland

**Keywords:** C82, L16, L81

## Abstract

Die Auswirkungen der Pandemie sind in allen Wirtschaftsbereichen spürbar. Ein Bereich, der am stärksten durch Schließungen, Einschränkungen und ein verändertes Verbraucher:innenverhalten betroffen war und bleibt, ist der innerstädtische Einzelhandel. Tatsächlich wird an der Passantenfrequenz in ausgewählten Städten der zum Teil dramatische Rückgang an potenziellen Kund:innen deutlich. In den statistischen Umsatzzahlen zum gesamten Einzelhandel wird dies nicht adäquat abgebildet. Eine nähere Betrachtung innerstädtischer Einzelhandelsbereiche offenbart, dass trotz erhöhter Nutzung von E-Commerce-Kanälen der Umsatz noch unter dem Vor-Corona-Niveau liegt. Zudem profitieren einzelne Bereiche stark, während andere dramatisch eingebüßt haben. Durch somit ausgelöste Geschäftsaufgaben kann jedoch die Attraktivität der Innenstadt als Ganzes sinken, weshalb auch Bereiche, welche die Krise bisher meistern konnten, am Ende mit sinkenden Umsätzen konfrontiert sein könnten.

## Abstract (en)

The coronavirus pandemic affects all sectors of the economy. The sector hardest hit by closures, restrictions and a changing consumer behavior is the inner-city retail. In fact, the frequency of passers-by and, therefore, potential customers in selected cities declined dramatically. The statistical sales figures that also include online retail and food retailers do not adequately represent the negative effects. A closer look at inner-city retail areas reveals that despite increased use of e-commerce channels, sales are still below the pre-corona level. Also, some areas of inner-city retail benefit, while others have lost dramatically. Therefore, triggered business failures can negatively affect the attractiveness of downtown as a whole and correspondingly, the areas that have been able to master the crisis in inner cities will also lose customers and turnover.

Die Coronapandemie dauert mittlerweile seit mehr als zwei Jahren an. Bisher hat sie für die gesamte deutsche Volkswirtschaft zu ökonomischen Verlusten von rund 350 Mrd. Euro geführt (Grömling, 2022). Zudem hat sich der wirtschaftliche Ausblick durch das Andauern der Pandemie mittlerweile wieder eingetrübt (Brautzsch et al., 2021). Somit ist davon auszugehen, dass die ökonomischen Verluste noch weiter zunehmen werden. Der innerstädtische Einzelhandel war und ist dabei besonders stark von der Pandemie betroffen. Er war mit Schließungen und Einschränkungen, die beispielsweise nur den Zugang von Genesenen und Geimpften erlauben (2G), konfrontiert, die insbesondere auch den umsatzstarken Zeitraum um Weihnachten beeinflusst haben (Goecke und Rusche, 2021). Diese Schließungen und Einschränkungen werden kontrovers diskutiert. Bei den gravierenden Auswirkungen der Pandemie insgesamt herrscht jedoch weitestgehend Einigkeit. Als aktueller Beleg für diese beiden Umstände dient der offene Brief der großen Lebensmitteleinzelhändler Aldi Nord, Aldi Süd, Edeka, Rewe sowie der Schwarz Unternehmenstreuhand, zu der insbesondere Kaufland und Lidl gehören, an mehrere Bundesminister:innen und Ministerpräsident:innen (Deutsches Verbändeforum, 2022). Dieser Brief ist insbesondere deshalb erwähnenswert, weil die genannten Händler durchaus ihre Umsätze in der Pandemie steigern konnten (Goecke und Rusche, 2021). In diesem Brief heißt es:

> „Wir teilen mit dem stationären Nonfood-Handel dessen tiefe Existenzsorgen und sehen die Gefahr von tausenden Schließungen insbesondere inhabergeführter Geschäfte und deren verheerende Wirkung auf unsere Innenstädte, die Standort-Agglomerationen und mithin den stationären Einzelhandel in Deutschland als Ganzes" (Deutsches Verbändeforum, 2022).

Vor diesem Hintergrund werden hier die Auswirkungen auf die Innenstädte quantitativ untersucht. Diese Untersuchung beruht auf zwei Säulen. Die erste Säule besteht in der Auswertung von Passantendaten in ausgewählten Innenstädten von deutschen Großstädten. Diese Säule ermöglicht es insbesondere, die Auswirkungen der Coronabeschränkungen auf die Kundenfrequenz zu schätzen. Die zweite Säule bildet die Auswertung von Daten des

**Dr. Christian Rusche** ist Economist für Industrieökonomik und Wettbewerb beim Institut der deutschen Wirtschaft in Köln.

Statistischen Bundesamts zum Umsatz im Einzelhandel. Mit Hilfe dieser Daten kann auch die Wirkung auf den Umsatz näher analysiert werden, die mit der Veränderung in der Frequenz einhergeht.

### Umsatz im Einzelhandel und Passantenfrequenz

In der Abbildung 1 sind die beiden empirischen Analysestränge dargestellt. Die Daten zur Zahl an Passant:innen stammen von der Firma Hystreet (hystreet.com). Hystreet hat in zahlreichen Innenstädten in Deutschland, Österreich, der Schweiz, Dänemark, den Niederlanden und Luxemburg Kameras installiert, die messen, wie viele Passant:innen den jeweiligen Abschnitt passieren. Für eine Passantenfrequenz von bis zu 500 Personen pro Minute gibt Hystreet eine Genauigkeit der Messung durch die Kameras von 99 % an. Die aus diesen Messungen resultierenden Werte für die Zahl an Passant:innen werden auf stündlicher Basis für die jeweiligen Zählstellen ausgewiesen. Die auf Tagesbasis aggregierten Werte für die deutschen Innenstädte wurden für die vorliegende Analyse verwendet.

Um eine Einschätzung der Aktivitäten in den Innenstädten während der Coronapandemie im Vergleich zu der Zeit vor der Pandemie zu haben, gehen die kompletten Jahre 2019, 2020 und 2021 in die Analyse ein. Dabei kommen nur Zählstellen in deutschen Innenstädten zum Einsatz, die für diese drei Jahre vollständige Daten aufweisen. Als vollständig werden Datensätze angesehen, bei denen im gesamten Betrachtungszeitraum maximal 20 Tageswerte fehlen. So ist gewährleistet, dass für jeden Monat immer mindestens ein Drittel gemessene Werte vorliegen. Einzelne Werte können fehlen, weil temporäre Störungen eine Messung nicht zulassen. Hierzu kann beispielsweise ein Ausfall der Kamera gehören oder eine versperrte Sicht der Kamera durch eine Baustelle. Auch Tage, für die die Passantenzahl mit einem Wert von Null angegeben ist, werden als fehlender Wert interpretiert. Sollten vereinzelt Werte fehlen, werden die entsprechenden Datenlücken unter Verwendung des Kalmanfilters imputiert (Steffen und Bartz-Beielstein, 2017). Diesem Ansatz folgend gehen in die Auswertung insgesamt 16 deutsche Städte mit insgesamt 27 Zählstellen ein. Diese Städte sind Berlin, Bonn, Düsseldorf, Frankfurt am Main, Freiburg, Hamburg, Hannover, Köln, Leipzig, Mainz, Mannheim, München, Münster, Nürnberg, Stuttgart und Wiesbaden. Die Hystreet-Daten besitzen die Einschränkung, dass sie nur an selektiven Abschnitten in ausgewählten (Groß-)Städten erhoben werden. Somit kann beispielsweise die Entwicklung in Randlagen und im ländlichen Raum nicht abgebildet werden. Dennoch bieten sie einen ersten Eindruck über die aufgetretenen Effekte.

Abbildung 1

**Umsatz im Einzelhandel sowie Zahl Passant:innen in den Innenstädten**

Absolut (Passant:innen) bzw. Umsatz in jeweiligen Preisen mit 2015 = 100

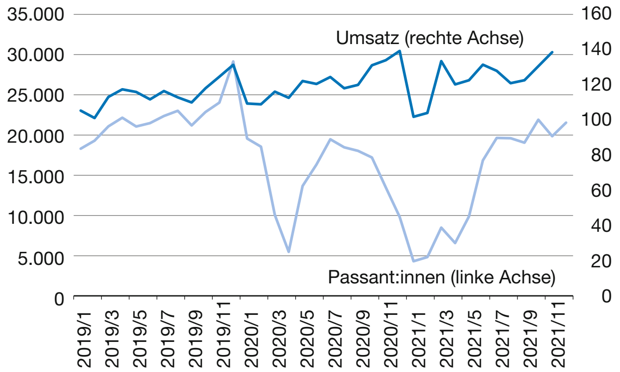

Quellen: Hystreet (2022); Statistisches Bundesamt (2022b); eigene Darstellung.

In der Abbildung wird anhand der Zahl Passant:innen des Jahres 2019 die typische Saisonalität im Einzelhandel deutlich (Goecke und Rusche, 2021). So steigt der Umsatz in der Regel bis zum Dezember tendenziell an, um danach im Januar einen Abfall und Tiefpunkt zu erleben. Konkret konnten für die Zählstellen in den genannten Städten im Januar 2019 rund 18,3 Mio. Passant:innen gezählt werden. Im Februar 2019 waren es bereits 19,3 Mio. In den Monaten danach wurde die Marke von 20 Mio. zum Teil deutlich überschritten. Das Maximum 2019 bildet der Dezember mit rund 29,1 Mio. Passant:innen. Danach fällt die Zahl der Passant:innen nahezu kontinuierlich bis auf rund 5,5 Mio. im April 2020, dem Höhepunkt des ersten Lockdowns. Durch die Lockerungen der Coronamaßnahmen stieg der Wert im Folgenden auf 19,5 Mio. im Juli 2020 an, ohne jedoch die entsprechenden Werte des Vorjahres zu erreichen. Danach fiel die Passant:innenzahl erneut bis auf einen Tiefstwert von 4,3 Mio. im Januar 2021 während des zweiten Lockdowns. Anschließend nahm die Frequenz zum Teil deutlich zu. Im Oktober 2021 konnte zudem mit 21,9 Mio. wieder die Marke von 20 Mio. überschritten werden. Die Werte im November (rund 19,9 Mio.) und Dezember (21,5 Mio.) waren jedoch bereits wieder geringer. Ursächlich dafür dürften die Zunahme des Infektionsgeschehens und 2G-Beschränkungen in bestimmten Bereichen des Handels sein (HDE, 2022).

Die in der Abbildung dargestellten Umsätze im Einzelhandel insgesamt ohne den Handel mit Kraftfahrzeugen gemäß Monatsstatistik des Statistischen Bundesamts (2022b) zeigen, dass diese nicht so stark schwanken wie die Passantenzahlen und zudem in der Pandemie sogar tendenziell zugenommen haben. Dargestellt sind die Originalwerte in jeweiligen Preisen, wobei das Statistische

Bundesamt die Werte auf das Jahr 2015 normiert hat (2015 = 100). Die Werte geben somit den Prozentwert des Umsatzes im Vergleich zu einem durchschnittlichen Monat des Jahres 2015 an. Den aktuellen Rand bilden die Werte für November 2021. Auch in den Umsätzen 2019 sind der Tiefpunkt zu Beginn eines Jahres und der Höhepunkt im Dezember zu erkennen. Konkret wurde im Februar 2019 der Tiefstwert von 101,1 erreicht. Im Vormonat waren es noch 105,3. Danach stieg der Wert tendenziell bis Dezember 2019 auf 131,1. In der Folgezeit sind die Werte ebenfalls gesunken. Mit 109,3 im Januar 2020 und 108,9 im Februar lagen die Tiefstwerte jedoch deutlich über den Werten des Vorjahres. Während der Lockdownmonate März und April stiegen die Umsätze zudem an. Dabei war der März 2020 umsatzstärker als der März 2019 (116,1 versus 113,1), während der April 2020 schwächer als der April 2019 verlief (112,6 versus 117,4). Danach stiegen die Umsätze bis auf 139,2 im Dezember 2020. Im Januar 2021 gingen die Umsätze auf 101,8 zurück. Somit sorgte der zweite Lockdown mit den Geschäftsschließungen im Einzelhandel ab 16.12.2020 auch bei den Umsätzen für einen Rückgang unter das Niveau von 2019 und 2020. Dennoch wurde der Tiefstwert vom Februar 2019 nicht unterschritten. Danach stiegen die Umsätze wieder an und erreichten mit 133,4 im März 2021 einen außergewöhnlich hohen Wert. Im weiteren Verlauf des Jahres schwankten die Umsätze zwischen 120,2 im April 2021 und 138,5 im November. Somit lagen die Umsätze im November 2021 über den entsprechenden Werten der beiden Vorjahre.

Zusammenfassend kann festgehalten werden, dass die Passantenzahlen im Betrachtungszeitraum sehr stark schwanken und deutlich von den Einschränkungen im Rahmen der Pandemiebekämpfung beeinflusst worden sind. Sie sind zudem auf außergewöhnliche Tiefstwerte abgesunken. Dies offenbart auch eine Betrachtung der Jahreswerte auf Basis der Hystreet-Daten. So wurden in den betrachteten Städten 2019 rund 266 Mio. Passant:innen gezählt. 2020 waren es nur noch rund 180 Mio. und 2021 lediglich rund 172 Mio. Die Umsätze im Einzelhandel insgesamt (ohne den Handel mit Kraftfahrzeugen) schwanken einerseits weit weniger stark und die Lockdownmaßnahmen schlagen sich hier kaum nieder. Diesen Umstand verdeutlicht exemplarisch der Dezember 2020. In diesem Monat wurden sehr hohe Umsätze bei gleichzeitig niedriger Passantenfrequenz in den Innenstädten verzeichnet. Über den Pandemiezeitraum hinweg haben die Umsätze im Einzelhandel zudem tendenziell zugenommen. Konkret sind die Umsätze im Einzelhandel im Jahr 2020 im Vergleich zu 2019 um 5,7 % gestiegen. 2021 lagen sie im Vergleich zu 2019 um rund 8,7 % höher (vgl. Tabelle 1). Die Gesamtumsätze bilden somit die Entwicklung in den Innenstädten nicht adäquat ab. Dies dürfte einerseits an einer Umsatzumlenkung beispielsweise zu Supermärkten, die nicht von Schließungen und 2G-Einschränkungen betroffen waren, liegen. Andererseits dürften auch erhebliche Umsätze im Einzelhandel aus dem Onlinehandel resultieren, der ebenfalls von der Pandemie profitierte (Rusche, 2021).

## Umsatzentwicklung im innerstädtischen Einzelhandel

Bevor die Umsätze in den Innenstädten näher betrachtet werden können, muss die Innenstadt zunächst statistisch abgegrenzt werden. Dazu wird auf das Vorgehen von Goecke und Rusche (2021) zurückgegriffen. Diese haben als erste Näherung die Abgrenzung mit Hilfe des Umsatzes je Unternehmen im Jahr 2018 gemäß der Jahresstatistik des Handels des Statistischen Bundesamts (2021) vorgenommen. Dieses unterteilt die Wirtschaftsbereiche gemäß der Klassifikation der Wirtschaftszweige, Ausgabe 2008 (WZ 2008; Statistisches Bundesamt, 2008). Die WZ-Klasse WZ08-47 umfasst dabei den Einzelhandel ohne den Handel mit Kraftfahrzeugen. Im Durchschnitt betrug der Umsatz je Unternehmen in dieser Klasse 1,8 Mio. Euro (Statistisches Bundesamt, 2021). Goecke und Rusche (2021) gehen von der Annahme aus, dass in den Innenstädten und Ortszentren die Fläche begrenzt ist, weshalb dort vor allem kleinere Unternehmen mit einem Umsatz von weniger als 1,8 Mio. Euro pro Jahr anzutreffen sind. Infolgedessen werden Supermärkte (WZ08-4711), Möbelmärkte (WZ08-47591) und Brennstoffhändler (WZ08-47991) nicht näher betrachtet. Im nächsten Schritt wurde die resultierende Kategorisierung in Innenstadt und Nicht-Innenstadt nochmals näher beleuchtet, um die Validität der Unterteilung zu erhöhen. Dies hat zur Folge, dass Apotheken[1] (WZ08-4773; 3,2 Mio. Euro pro Unternehmen), der Einzelhandel mit kosmetischen Erzeugnissen und Körperpflegemitteln (WZ08-4775; 5,5 Mio. Euro), der Handel mit Unterhaltungselektronik (WZ08-4743; 2,2 Mio. Euro) sowie der Sonstige Einzelhandel mit Waren verschiedener Art ohne Nahrungsmittel (WZ08-4719; 2,8 Mio. Euro) zusätzlich zur Innenstadt gezählt werden, weil Betriebe dieser Bereiche vor allem auch in den Innenstädten anzutreffen sind. Des Weiteren wurde der Bereich Einzelhandel mit Metallwaren, Anstrichmitteln sowie Bau- und Heimwerkerbedarf (WZ08-4752; 1,7 Mio. Euro) herausgerechnet, da die Innenstadt nicht (mehr) die Hauptdomäne dieses Bereichs sein dürfte. Abweichend zu Goecke und Rusche (2021) wurde hier auch der Bereich „Sonstiger Einzelhandel anders nicht genannt“ (nicht in Verkaufsräumen; WZ08-47999;

1 Apotheken weisen Besonderheiten im Vertrieb auf, wodurch dieser Bereich beispielsweise vom Handelsverband Deutschland (HDE) nicht zum klassischen Einzelhandel gezählt wird. Insofern ist die Einbeziehung nicht einheitlich.

Tabelle 1

**Umsatz im innerstädtischen Einzelhandel 2019 sowie dessen Veränderung 2020 und 2021 im Vergleich zu 2019**

Umsatz in Mio. Euro ohne Umsatzsteuer in jeweiligen Preisen sowie Veränderung in %

| WZ08-Klasse | Einzelhandelsgruppe | Umsatz 2019 in Mio. Euro | Veränderung Umsatz 2020 im Vergleich zu 2019 in % | Veränderung Umsatz 2021[1] im Vergleich zu 2019 in % |
|---|---|---|---|---|
| 4719 | Sonstige Waren verschiedener Art ohne Lebensmittel (z. B. Erotik-Shop, Warenhaus) | 11.626 | -11,9 | -16,2 |
| 472 | Nahrungsmittel usw. (z. B. Bäckerei, Fleisch, Fisch) | 28.870 | 3,8 | 6,1 |
| 474 | Kommunikations- und Informationstechnik | 19.851 | -2,3 | -14,9 |
| 4751 | Einzelhandel mit Textilien (z. B. Bettwaren und Tischwäsche) | 1.555 | -0,1 | -11,2 |
| 4753 | Vorhänge, Teppiche und Bodenbeläge, Tapeten | 2.862 | 11,7 | 1,5 |
| 4754 | Elektrische Haushaltsgeräte | 3.650 | 9,2 | 8,8 |
| 47592 | Keramische Erzeugnisse und Glaswaren | 626 | 6,9 | 0,7 |
| 47593 | Musikinstrumente und Musikalien | 693 | -4,7 | -8,9 |
| 47599 | Haushaltsgegenstände anders nicht genannt (z. B. Kinderwagen, Korbwaren) | 1.819 | 8,0 | 8,3 |
| 476 | Sportausrüstung, Verlagserzeugnisse und Spielwaren | 17.353 | 5,3 | 1,9 |
| | davon Fahrräder, Sport- und Campingartikel | 10.542 | 15,0 | 14,0 |
| 477 | Sonstige Güter in Verkaufsräumen (z. B. Apotheken, Schuhe, Lederwaren) | 153.752 | -4,5 | -0,6 |
| | davon Apotheken | 56.742 | 7,1 | 16,6 |
| 478 | Verkaufsstände und Märkte | 1.442 | 4,9 | 7,4 |
| Gesamt | Innenstadt | 244.099 | -2,4 | -1,3 |
| 47 | Einzelhandel (ohne Handel mit Kraftfahrzeugen) insgesamt | 595.418 | 5,7 | 8,7 |

[1]Januar bis November.

Quellen: Statistisches Bundesamt (2022a); eigene Berechnungen auf Basis Statistisches Bundesamt (2022b; 2022c)

352.000 Euro) nicht zur Innenstadt gezählt, da dieser insbesondere Haustürgeschäfte, Automatenverkäufe und Tiefkühlheimservice umfasst, die überwiegend nicht zur Innenstadt zählen dürften. Diese Unterteilung ist insgesamt zwar nicht trennscharf, dennoch bietet sie eine erste Näherung, um die Entwicklung in den Innenstädten statistisch abgrenzen zu können.

Die Ergebnisse dieser Unterteilung sind in Tabelle 1 dargestellt. Gemäß der Jahresstatistik des Handels des Statistisches Bundesamts (2022a) konnte 2019 in den Innenstädten ein Umsatz ohne Umsatzsteuer von rund 244,1 Mrd. Euro verzeichnet werden. Dieser beinhaltet bereits Umsätze im Onlinehandel der entsprechenden Unternehmen. Werden die Umsatzwerte der Jahresstatistik mit Hilfe der Monatsstatistik (Statistisches Bundesamt, 2022b; 2022c) fortgeschrieben, kann die Entwicklung der Umsätze in den Innenstädten nach Wirtschaftsbereichen genauer analysiert werden. So sank der Umsatz 2020 im Vergleich zu 2019 in jeweiligen Preisen um 2,4 %. Im Jahr 2021 (Januar-November) war der Umsatz im Vergleich zu 2019 um 1,3 % niedriger. Somit konnte 2021 der Rückgang im Vergleich zu 2019 verringert werden. Die Vor-Corona-Werte konnten jedoch nicht erreicht werden. Da die dargestellten Werte in jeweiligen Preisen angegeben werden, sind Preissteigerungen durch Inflation bereits enthalten. Real, d. h. nach Abzug der Inflation, dürfte der Rückgang noch deutlicher sein. Zudem ist in den Werten die verstärkte Nutzung von Onlinekanälen durch stationäre Händler enthalten (HDE, 2020). Die tatsächlichen Umsätze in den stationären Niederlassungen dürften somit nochmals niedriger liegen.

Zusätzlich offenbart die Betrachtung der einzelnen Einzelhandelsbereiche, dass sich die Entwicklung je nach Bereich stark unterscheidet. Die Gesamtbetrachtung der Innenstadt ließ dies so nicht erkennen. So lag der Umsatz bei Apotheken 2021 um fast 17 % oberhalb des Niveaus von 2019. Ebenfalls profitiert hat der Handel mit Fahrrädern, Sport- und Campingartikeln (Umsatz 2021 14 % über dem Niveau von 2019), der Handel mit elektrischen Haushaltsgeräten (+8,8 %) und der Handel mit „Haushaltsgegenstände anders nicht genannt" (z. B. Kinderwagen, +8,3 %). Demgegenüber stehen jedoch innerstädtische Bereiche, die deutliche Umsatzeinbußen hinnehmen mussten. Besonders deutlich war der Rückgang in den Bereichen „Sonstige Waren verschiedener Art ohne Lebensmittel" (z. B. Erotik-Shop, Kauf- und Warenhaus,

-16,2 %) sowie Kommunikation- und Informationstechnik (-14,9 %). Der letztgenannte Bereich überrascht dabei, da im Zuge von Homeoffice und sogenannten Stay-At-Home-Trends eine gegenteilige Entwicklung zu vermuten wäre. Dies dürfte darauf zurückzuführen sein, dass die entsprechenden Umsätze zu etablierten Onlinehändlern verlagert wurden und die stationären Händler:innen durch eigene E-Commerce-Angebote den Rückgang beispielsweise infolge von Einschränkungen nicht kompensieren konnten.

## Fazit

Zusammenfassend wird deutlich, dass der Vergleich der Passantenzahlen mit der Umsatzentwicklung im gesamten Einzelhandel keinen Zusammenhang zwischen diesen Bereichen vermuten lässt. So hat die Passantenfrequenz in den verfügbaren Städten infolge der Pandemie deutlich abgenommen, während der Umsatz insgesamt sogar zugenommen hat. Eine Bereinigung des gesamten Einzelhandels um Bereiche, deren wirtschaftliche Haupttätigkeit mutmaßlich nicht (mehr) in der Innenstadt liegt, offenbart ein realistischeres Bild. Dabei wird deutlich, dass trotz eines leichten Wachstums der Umsätze 2021 im Vergleich zum Jahr 2020 selbst nominal das Vor-Corona-Niveau noch nicht wieder erreicht werden konnte. Da auch die innerstädtischen Händler:innen vermehrt E-Commerce-Kanäle nutzen, dürften die eigentlichen Umsätze in den Innenstädten tatsächlich geringer ausfallen. Des Weiteren divergieren auch innerhalb der innerstädtischen Bereiche die Entwicklungen stark. So konnten einzelne Bereiche wie Apotheken deutlich an Umsätzen zulegen, während andere Bereiche mit erheblichen Umsatzeinbußen konfrontiert waren. Somit hat die Pandemie den Einzelhandel als Ganzes nicht negativ beeinflusst. Die Innenstädte sind jedoch zum Teil erheblich negativ betroffen, was an den Passantenzahlen und den Umsatzrückgängen einzelner Bereiche deutlich wird. Durch vermehrte Geschäftsaufgaben in bestimmten Einzelhandelsbereichen infolge der Pandemie sinkt die Attraktivität der Innenstädte zunehmend, wodurch auch die Bereiche, die bisher gut durch die Pandemie gekommen sind, mit einer negativen Geschäftsentwicklung konfrontiert sein werden. Einerseits haben sich Konsument:innen an die Nutzung des Onlinehandels gewöhnt und andererseits kann infolge einer niedrigen Attraktivität auch die Passantenfrequenz und damit der (potenzielle) Umsatz weiter sinken. Somit sind die dargelegten Entwicklungen lediglich eine Momentaufnahme, die einer weiteren Beobachtung und Analyse bedarf.

**Title:** ***The Impact of the Corona Pandemic on Retail in Inner Cities***
